# Core-shell structured nanomaterials in dual-modal magnetic resonance imaging guided antitumor effect via combined treatment

**DOI:** 10.3389/fchem.2026.1831059

**Published:** 2026-06-16

**Authors:** Wei Liu, Chengxin Liu, Longhai Jin, Jianqiu Wang, Jiale Tian, Tianqi Zhang, Jianhua Liu, Yinghui Wang, Shuyan Song

**Affiliations:** 1 Department of Radiology, The Second Hospital of Jilin University, Changchun, China; 2 State Key Laboratory of Rare Earth Resource Utilization, Changchun Institute of Applied Chemistry Chinese Academy of Sciences, Changchun, China

**Keywords:** breast cancer, CDT, MRI, PTT, theranostic

## Abstract

Accurate detection and localization of early-stage breast cancer represent the cornerstone for improving cancer cure rates and extending patient survival. However, selecting therapeutic regimens characterized by high efficacy and minimal side effects remains a formidable clinical challenge. Combining these two goals is now feasible with the innovation and progress of nanomaterials. Herein, we designed and synthesized a novel nanomaterial PDA@Fe-BTC@ MnO_2_ nanoparticles (PFMPP NPs) for breast cancer. The MnO_2_ and Fe-BTC units confer tumor-microenvironment (TME)-responsive T_1_/T_2_ dual-mode magnetic resonance imaging (MRI)capability. For therapy, the Fe-BTC structure mediates chemodynamic therapy (CDT) via the Fenton-like reaction, generating cytotoxic reactive oxygen species (ROS). Simultaneously, the MnO_2_ component catalyzes H_2_O_2_ decomposition to relieve tumor hypoxia and consumes reduced glutathione (GSH) to protect ROS from clearance. Furthermore, PFMPP NPs exhibit strong photothermal conversion under 808 nm near-infrared light, permitting direct tumor ablation via photothermal therapy (PTT). The localized heating also enhances the Fenton-like reaction, yielding more ROS and thereby enabling enhanced combined therapeutic efficacy. This integrated strategy provides a feasible approach for theranostic applications in breast cancer.

## Introduction

Breast cancer has emerged as the most commonly diagnosed cancer and a leading cause of cancer-related mortality among women worldwide, representing a severe and growing global health challenge. According to the recently Global Burden of Disease (GBD) 2023 Study, there were approximately 2.30 million new incident cases and 764,000 deaths attributable to female breast cancer in 2023, resulting in an enormous 24.1 million disability-adjusted life years (DALYs) lost globally. Despite the rapid development of modern medicine, it is still difficult to overcome malignant tumors with existing methods (Collaborators, 2026; Feng et al., 2019; Sharkey and Cook, 2025; Terashima et al., 2025). In clinical practice, several modalities are widely used for cancer diagnosis and prognosis, including ultrasound, mammography, computed tomography (CT), positron emission tomography (PET-CT), tumor biomarkers, and pathological biopsy. However, these approaches suffer from inherent limitations such as low resolution, ionizing radiation, invasiveness, poor specificity, or low sensitivity. Although emerging nanoscale technologies and machine learning methods have shown promising potential, they still face obstacles including complex pre-calibration procedures and high dependence on large experimental datasets, respectively, restricting their clinical application (Arravalli et al., 2025; Marcuello et al., 2025). In contrast, magnetic resonance imaging (MRI) offers superior soft-tissue contrast, multi-parameter imaging, and non-invasiveness, making it a more feasible and reliable tool for cancer diagnosis and prognosis. When subjected to an external magnetic field, nuclei require varying times to relax from an excited state back to equilibrium, depending on their specific molecular environment. This time is referred to as relaxation time, which is intuitively reflected in the varied signal intensity of the images and can be used to diagnose tumors or other diseases (Yang et al., 2025; Zhao et al., 2023). Longitudinal relaxation time T_1_ can more directly reflect the physiological structure of tissue, and transverse relaxation time T_2_ is better at finding the location and internal characteristics of lesions. However, the diagnostic sensitivity and specificity of routine MRI are still limited without the assistance of contrast agents. Nevertheless, most of the current commercial contrast agents are T_1_-weighted single model imaging agents, which lose a large amount of valid information (Barandov et al., 2016; Dai et al., 2018; Li J. et al., 2018). Therefore, designing and fabricating nanoprobes with good biocompacity for MR T_1_/T_2_-weighted dual-modal imaging is highly desirous for efficient and accurate diagnosis.

The subsequent targeted individual treatment is equally important once the tumor is diagnosed and precisely located. The most common tumor treatments are surgical resection, chemotherapy, and radiotherapy. However, some drawbacks are associated with these treatment measures, including difficulty avoiding damage to normal tissues or adverse consequences such as systemic toxicity and drug tolerance (Li et al., 2020; Shi and Sadler, 2020; Singhal, 2016). With the coordinated development of nanotechnology and medicine, specific treatments for the tumor microenvironment (TME) have gradually garnered the attention of researchers (Liu et al., 2023; Tang et al., 2017; Trachootham et al., 2009; Wang et al., 2019; Yang et al., 2023). Due to the accumulation of lactic acid and overexpression of superoxide dismutase at the tumor site, a weak acidity, elevated H_2_O_2_, high glutathione (GSH) and hypoxia environment is formed (Arneth, 2019; Dai et al., 2017; Yang and Gao, 2017). Chemodynamic therapy (CDT) is a specific therapy based on TME, using Fenton or Fenton-like reactions to produce reactive oxygen species (ROS), leading to apoptosis. Because the Fenton reaction’s efficiency limits the success of tumor therapies, additional assistance is needed to improve the reaction efficiency. Some studies have proved that increasing the temperature of the neoplastic tissue can improve the Fenton reaction efficiency (Shi et al., 2025; Zhang et al., 2020; Zhao et al., 2022). Therefore, the combination of photothermal therapy (PTT) and CDT can significantly improve the killing of tumor cells. The advantage of PTT is that it can use photothermal conversion materials to convert near-infrared light (NIR) into heat, directly killing tumor cells with an increase in temperature. Cell death caused by high temperature does not have drug resistance, and by controlling the location of NIR exposure, it can also reduce damage to normal tissues, especially for breast cancer located on the body surface. However, current photothermal materials are mainly organic small molecule dyes or inorganic metal nanomaterials, such as indocyanine green (ICG), gold, and carbon nanomaterials (Huang et al., 2006; Wang et al., 2010; Yang et al., 2010; Yang et al., 2011). Organic dyes tend to bind to plasma proteins, accumulate in the liver, and are easily bleached under high temperatures or light, limiting their application prospects in tumor sites. On the other hand, though the photothermal properties of inorganic metal nanomaterials have been significantly improved, their long-term toxicity still needs to be carefully considered. Therefore, it is necessary to find safer and more stable photothermal materials to ensure the reliable efficacy of PTT (Hong et al., 2020; Li H. et al., 2018).

**SCHEME 1:**
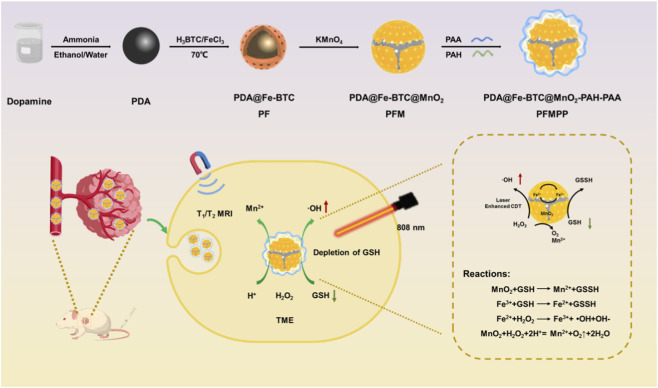
Schematic illustration of the application of PFMPP NPs.

In this work, we have designed and synthesized a poly (allylamine hydrochloride) (PAH) and Poly (acrylic acid) (PAA) co-modified PDA@Fe-BTC@MnO_2_-PAH-PAA nanoparticles (PFMPP NPs) for dual-modal MRI guided CDT and PTT synergistic treatment for breast cancer. Compared with the single-mode MRI, TME-responsive T_1_/T_2_-weighted dual-modal MRI can improve the accuracy and reliability of diagnosis (Shi et al., 2023; Zhang et al., 2024). Meanwhile, it can also perform as an anti-tumor drug, where PTT cooperates with enhanced CDT in TME. As shown in Scheme 1, MnO_2_ on the surface of PFMPP NPs can catalyze O_2_ generation in high H_2_O_2_ concentration of in the TME, alleviating local hypoxia which can reduce the risk of tumor metastasis. Besides, MnO_2_ can decompose into Mn^2+^ under acidic and high GSH conditions at the tumor site, enabling the TME -responsive T_1_-weighted MRI. Then, the exposed Fe-BTC component can be a negative contrast agent for T_2_-weighted imaging, and the surface of Fe-BTC can release Fe^3+^ slowly. It has been confirmed that Fe^3+^ generates •OH (the most toxic ROS) through Fenton-like reaction in the TME, with high levels of acid, MnO_2_, and GSH. Polydopamine (PDA), serving as the core of PFMPP NPs, exhibits excellent biocompatibility due to its precursor being a natural neurotransmitter in organism. Meanwhile, PDA not only functions as an efficient photothermal conversion medium upon 808 nm laser irradiation, but also remarkably improves •OH generation efficiency through enhancement of Fenton-like catalytic effects. Thus, PFMPP NPs have promising applications in tumor TME-responsive T_1_/T_2_ dual-modal MRI-guided PTT and CDT antitumor effect via combined treatment for breast cancer.

## Methods

### Synthesis of PDA

40 mL of ethanol and 100 mL of DI water were vigorously stirred followed by the addition of 3 mL of ammonia solution. After 10 min, 0.5 g of dopamine hydrochloride was added to the reaction system. PDA were finally obtained after stiring at room temperature for 24 h, then centrifugated and washed with DI water three times.

### Synthesis of PDA@Fe-BTC

5 mg PDA were added to an ethanolic solution of FeCl_3_·6H_2_O (1 mL, 0.5 M) and stirred for 1 h and collected by centrifugation. To prepare PDA@Fe-BTC nanoparticles (PF NPs), a dispersion of the product of the above in ethanol was mixed with H_3_BTC and kept at 70 °C for 40 min. In order to increase the loading of Fe^3+^, the above step was repeated four times and washed with ethanol three times. The PF NPs were mixed into ethanol and stored at room conditions.

### Synthesis of PDA@Fe-BTC@MnO_2_-PAH-PAA

PF NPs were centrifuged and dispersed in DI water (1 mg mL^−1^) and then mixed with MES aqueous solution (0.1 M, pH 6.0) by ultrasonication for uniform distribution. Then, the KMnO_4_ aqueous solution (0.05 M, 1 mL) was added with ultrasonication for 6 min, and the PDA@Fe-BTC@MnO_2_ nanoparticles (PFM NPs) were washed with DI water three times. Subsequently, PFM NPs (1 mg mL^−1^) were sequentially mixed with PAH (0.1 mg mL^−1^) and PAA (0.1 mg mL^−1^) solutions for 6 h each, and finally PDA@Fe-BTC@MnO_2_-PAH-PAA nanoparticles (PFMPP NPs) were stored in DI water.

### Measurement of extracellular •OH

PFMPP NPs (Fe^3+^, 0.2 mM) and H_2_O_2_ (1 mM) were mixed with a pH-gradient PBS buffer system (5.0, 6.5, and 7.4). PTA (20 μL, 25 μg mL^−1^) was used as the probe to detect the presence of •OH. Using 315 nm as the excitation wavelength, PTA combined with •OH shows a pronounced emission peak at 425 nm. Then, the earlier-mentioned solutions were subjected to NIR (808 nm, 1.0 W cm^-2^). The corresponding 425 nm fluorescence was measured by a fluorescence spectrometer. Next, different concentrations (Fe^3+^, 0–0.8 mM) of PFMPP NPs was mixed with PBS buffer (pH 6.5 ± 0.2, 1 mM H_2_O_2_), and subsequently, fluorescence probe PTA was added to detect the 425 nm emission. In the third set of experiments, different concentrations of H_2_O_2_ (0–8 mM) were added in PBS buffer solution (pH 6.5) with PFMPP NPs (Fe^3+^, 0.2 mM), followed by the addition of PTA as a fluorescence probe to detect the emission peak at 425 nm.

### Measurements of extracellular O_2_ generation

The O_2_ concentration was detected by a dissolved oxygen meter. 40 μL H_2_O_2_ (1 M) was added to an aqueous solution of PFMPP NPs (20 mL, 1 mM) and the oxygen concentration was measured every 10 s.

### Photothermal effect and thermal stability

The aqueous solutions of PFMPP NPs were subjected to NIR (808 nm, 0.7, 1.0 and 1.3 W cm^−2^) for 10 min, and their corresponding temperatures were recorded per 30 s. Further, the thermal stability of PFMPP NPs was investigated. The earlier-mentioned PFMPP NPs solutions (100 μg mL^−1^) was irradiated by 808 nm laser (1.0 W cm^−2^) for 10 min, cooled naturally to room temperature, and then the temperature was recorded every 30 s. The above experiment was repeated 4 times.

### Cytotoxicity of PFMPP NPs

L929 and 4T1 cells were respectively cultured for 24 h (37 °C, 5% CO_2_). The different concentrations of PFMPP NPs (0, 12.5, 50, 100, and 200 μg mL^−1^) were added to the plate and incubated. Before recording the 450 nm emission, the cells were washed and followed by adding CCK-8 solution.

### *In vitro* photothermal therapy

4T1 cells were cultured under standard conditions. Then, the as above concentrations PFMPP NPs were co-cultured with cells. After 24 h, the cells were washed and subjected to NIR (808 nm, 1.0 W cm^−2^). The cell viability was tested by CCK-8 assay.

### *In vitro* chemodynamic therapy

4T1 cells were cultured under standard conditions, and as above concentrations of PFMPP NPs and H_2_O_2_ (1 mM) were added and co-cultured with cells. After 24 h, the cell viability was tested by CCK-8 assay.

### *In vitro* photothermal-assisted chemodynamic therapy

4T1 cells were cultured under standard conditions, and as above concentrations of PFMPP NPs and H_2_O_2_ (1 mM) were added and co-cultured with cells for 24 h. The cells were washed and subjected to NIR (808 nm, 1.0 W cm^−2^). The cell viability was tested by CCK-8 assay.

### AM/PI Co-staining

4T1 cells were co-cultured with PFMPP NPs (200 μg mL^−1^) under standard conditions, and these cells were randomly incubated with or without H_2_O_2_ (1 mM) for 24 h. After subjecting to NIR (808 nm, 1.0 W cm^−2^), the cells were co-stained with AM/PI mixed solution for 30 min and collected their photos by fluorescence microscopy.

### Detection of intracellular •OH

4T1 cells were co-cultured with PFMPP NPs (200 μg mL^−1^) under standard conditions, and these cells were randomly incubated with or without H_2_O_2_ (1 mM) for 24 h. Afterward, the cells were subjected to NIR (808 nm, 1.0 W cm^−2^) for 10 min and stained with DCFH-DA for 30 min. The cells were washed again and collected their photos by fluorescence microscopy.

### Detection of intracellular GSH

The GSH levels were detected by GSH assay kits (Jiancheng Institute of Bioengineering, A006-2-1). 4T1 cells were cultured as the above methods and added different concentration of PFMPP NPs (0, 25, 50, 100 μg mL^−1^). After the pre-treatment, 4 groups of cells were subjected to cell lysis and operated one by one according to the steps respectively. Then, the samples were detected and evaluated according to the methods in GSH assay kits.

### *In vivo* therapy and photothermal imaging

Female BALB/c mice were selected for establishing 4T1 orthotopic breast cancer models and operations comply with ethical requirements. Randomly select 32 tumor-bearing mice and divide them into four groups on average: (a) control group; (b) NIR group; (c) PFMPP NPs group; and (d) PFMPP NPs + NIR group. The (a) and (b) groups were both injected with PBS during the treatment. Group (b) was treated by NIR (808 nm, 1.0 W cm^−2^). The (c) and (d) groups were injected with PFMPP NPs (100 μL, 1 mg mL^−1^) via tail vein every 2 days, and the (d) group was irradiated by NIR irradiation (808 nm, 1.0 W cm^−2^). The weight and tumor size (length × 1/2 width^2^) of mice were recorded every 2 days. Infrared thermal cameras were used to record the temperature of tumor locations in (b) and (d) groups every 30 s.

### Biodistribution of PFMPP NPs

PFMPP NPs were intravenously administered to 5 tumor-bearing mice at a proper dose (100 μL, 1 mg mL^−1^). The important organs (heart, liver, spleen, lungs, and kidneys) and tumors were resected, and their weights were recorded. The resected tissues were then put into 3 mL aqua regia for 7 days, and the amount of Mn^2+^ was determined by ICP.

### H&E and TUNEL staining

After the initial treatment, one mouse was randomly selected from each of the four groups, euthanized, and subjected to tumor tissue excision followed by H&E and TUNEL staining. Another group of healthy female Balb/C mice was only injected with PFMPP NPs (100 μL, 1 mg mL^−1^). After 30 days, their important organs were collected for H&E staining.

### *In vitro* MRI

The dual-mode imaging of PFMPP NPs was performed by an Ingenia 3.0 T CX MRI machine (T_1_-weighted: TR 513 ms, TE 8 ms, Flip Angle 100°, T_2_-weighted: TR 3.0 s, TE 81 ms, Flip Angle 90°), with the concentration Mn (0–0.45 mM) and Fe (0–3.2 mM). The Ingenia 3.0 T CX MRI can measure the relaxation time through the software. The 4T1 cells were incubated with PFMPP NPs (pH 7.4) for 24 h under 37 °C without GSH. Then, the cells were washed, digested, and prepared in agarose gel for MRI analysis.

### *In vivo* MRI

The tumor-bearing mice were subjected to dual-modal imaging performed by an Ingenia 3.0 T CX MRI machine (T_1_-weighted: TR 1.9 s, TE 18 ms, Flip Angle 90°, T_2_-weighted: TR 3.6 s, TE 110 ms, Flip Angle 90°). MRI images before injection were collected as control. Then the PFMPP NPs (100 μL, 2 mg mL^−1^) with PBS solution under pH 7.4 were intravenously administered to the mice. The MRI images of mice were again collected after 24 h maintained at room temperature. Acquisition parameters were generated by the Ingenia 3.0 T CX MRI default.

## Results and discussion

### Synthesis and characterization of PFMPP NPs

**FIGURE 1:**
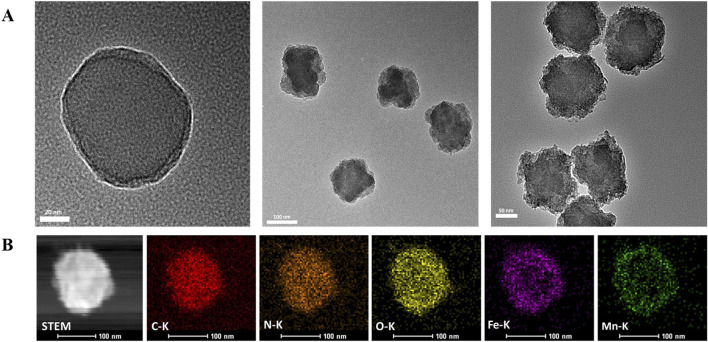
**(A)** TEM images of PFMPP NPs. **(B)** HAADF-STEM image and elemental mapping of C, N, O, Fe and Mn.

The formation of PFMPP NPs includes four steps. First of all, as shown in Scheme 1, the PDA sphere was synthesized following a literature-reported protocol to enable subsequent functionalization (Simon, 2000). In the next step, FeCl_3_·6H_2_O and H_3_BTC were chosen as precursors to fabricate the Fe-BTC shell. Fe-BTC is a derivative structure of MIL-100 (Fe), which commonly used for drug delivery, and has been proven to have good biocompatibility (Zhu et al., 2023). The MnO_2_ shell was also coated further on the outside of thus prepared Fe-BTC shell by adding KMnO_4_ into the weakly acidicbuffer solution under ultrasonication. Finally, PAH and PAA were successfully coated on the surface of Fe-BTC shell as two polymers with good water solubility to improve the biocompatibility of PFM NP. The transmission electron microscope (TEM) image exhibits that PDA core has a sphere-like shape with a diameter under 100 nm. After the PDA spheres are coated with Fe-BTC and MnO_2_, the diameter of the resulting nanoparticles is significantly larger than that of the pristine PDA spheres, and the surface of the nanoparticles changes from smooth to rough (Figure 1A). These structural changes confirm the successful synthesis of the PFMPP NPs. As demonstrated in Figure 1B, the final product PFMPP NPs, exhibits monodisperse nanostructure with a mean particle size of 150 nm. Meanwhile, the distribution of C, N, O, Fe, and Mn in PFMPP NPs was confirmed by TEM-EDS elemental mapping (Figure 1B), proving that this complex core-shell structure consists of all these elements. In Supplementary Figure S1, the morphology changes and size variation of PFMPP NPs were also very evident by scanning electron microscopy (SEM) (Supplementary Material).

**FIGURE 2:**
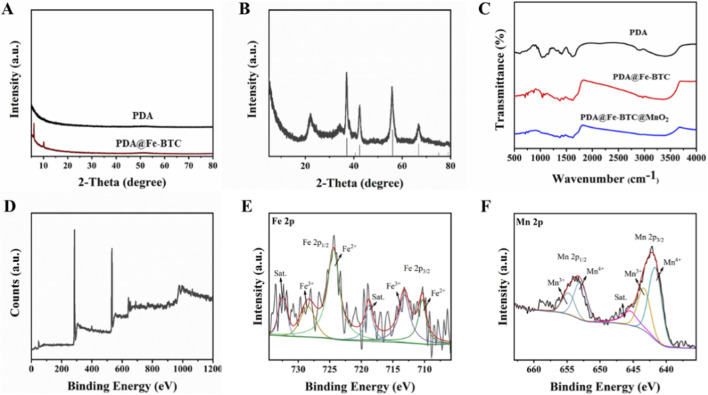
**(A)** XRD patterns of PDA, PDA@Fe-BTC and **(B)** PFMPP NPs. **(C)** FTIR spectra of PDAs, PDA@Fe-BTC and PFMPP NPs. **(D)** XPS spectrum of PFMPP NPs. **(E)** and **(F)** The spectra of Fe, Mn and their fitting curves.

As the core component, PDA spheres exhibited no characteristic peaks in X-ray diffraction (XRD) analysis (Figure 2A). However, after coating Fe-BTC on the PDA surface, two characteristic peaks appeared in the 1°–10° range of the XRD pattern, and the positions of these two peaks were roughly consistent with those reported in the literature. Subsequently, MnO_2_ shell was coated on the Fe-BTC surface using KMnO_4_, and the presence of MnO_2_ was further confirmed by XRD analysis (PDF #30-0820) (Hu et al., 2016). Nevertheless, due to the loose and porous structure of Fe-BTC, its crystallinity is relatively low, making it difficult to observe the characteristic peaks in the 1°–10° range in the XRD pattern of PFMPP NPs (Figure 2B). The FT-IR investigations also revealed the successful synthesis of PDA and subsequent layer-by-layer coating with Fe-BTC and MnO_2_. Some bands of PDA and PDA@BTC cannot be observed after coated by MnO_2_ (Figure 2C).

Subsequently, the X-ray photoelectron spectroscopy (XPS) was employed to conduct an in-depth analysis of the chemical composition of PFMPP NPs. The full XPS spectrum of this nanoparticle is presented in Figure 2D, which confirms the presence of Fe and Mn elements as well as their respective binding energies. As shown in Figure 2E, two peaks appear at binding energies of 724.3 eV and 710.3 eV, corresponding to Fe 2p_1_/_2_ and Fe 2p_3_/_2_ respectively. These bands confirm the presence of Fe^2+^, while the peaks at 728.0 eV and 713.2 eV are attributed to Fe^3+^. The coexistence of Fe^2+^ and Fe^3+^ in the material further ensures that the loose and porous Fe-BTC structure possesses the potential to undergo the Fenton-like reaction. Analysis of the Mn element confirmed that the binding energy at 642.1 eV corresponds to Mn 2p_3_/_2_, while the peak at 653.6 eV is attributed to Mn 2p_1_/_2_. This result indicates the presence of Mn^4+^, which is in agreement with published data (Figure 2F) (Zhang et al., 2014).

To enhance the *in vivo* applicability of PFMPP NPs, surface modification is necessary. Zeta potential analysis confirmed that the surface of MnO_2_ has negatively charged (Supplementary Figure S2); thus, positively charged PAH was selected for its modification to improve the aqueous stability of the PFM NPs. However, blood plasma contains a large number of negatively charged proteins, and nanoparticles with a positively charged surface are easily adsorbed by these proteins and deposit in parenchymal organs, such as the liver or spleen during blood circulation, which impairs the PFM NPs *in vivo* functionality. Therefore, further modification of the nanoparticles with negatively charged PAA is essential. This modification not only further enhances the aqueous stability but also improves the biocompatibility of the nanoparticles. Zeta potential measurements confirmed the successful adsorption of PAH and PAA onto the nanoparticle surface. When the modified PFMPP NPs dispersed in DI water, PBS, or cell culture medium, they were demonstrated excellent dispersibility and showed no visible precipitation or agglomeration even after long-term storage at room temperature (Supplementary Figure S3). Meanwhile, dynamic light scattering (DLS) was used to track the particle size throughout the stepwise surface modification. All measured sizes fell within an appropriate range (Supplementary Figure S4).

### Enhanced *in vitro* CDT and PTT using PFMPP NPs

**FIGURE 3:**
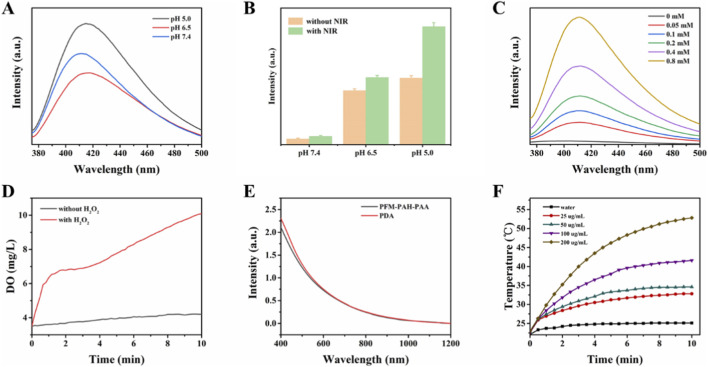
**(A)** The amount of •OH generation at different pH values. **(B)** Comparison of the •OH generation before and after laser irradiation. **(C)** Amount of •OH generated under different concentrations of PFMPP NPs. **(D)** Effect of PFMPP NPs catalysis the oxygen production. **(E)** UV-vis absorption curves of PDA and PFMPP NPs. **(F)** Photothermal heating profiles of PFMPP NPs at different concentrations under laser irradiation.

To assess the efficacy of PFMPP NPs to generate •OH radicals, we used the p-phthalic acid (PTA) as the ROS fluorescence probe. In Figure 3A, the observed increase of fluorescence intensity at 425 nm with the decreasing pH (from 7.4 to 5.0) confirms that an acidic environment accelerates the Fenton-like reaction. The results indicate that a very small amount of •OH is produced under normal pH 7.4, but the production of •OH significantly rises under the simulating tumor environment. Thus, PFMPP NPs demonstrate selectivity for the TME, thereby minimizing substantial damage to normal tissue. Next, the above solutions of different pH were irradiated by laser for 6 min. The corresponding fluorescence intensities before and after irradiation are shown in Figure 3B; Supplementary Figure S5A, demonstrating that laser irradiation promoted the generation of •OH. The production of •OH upon laser irradiation was even more under acidic conditions. Further, the effect of different concentrations of PFMPP NPs in the PBS solution was investigated. As anticipated, the results showed more •OH production with the increasing concentration of PFMPP NPs (Figure 3C). The initiation of the Fenton-like reaction relies on the reduction of inert ferric ions (Fe^3+^) to active ferrous ions (Fe^2+^). In the TME, GSH acts as a potent reducing agent, donating electrons to Fe^3+^ and converting to its oxidized dimeric form (GSSG), simultaneously regenerating Fe^2+^. This reduction step is crucial because it not only depletes the endogenous antioxidant reserves of the tumor but also sustains the catalytic cycle necessary for generating •OH. Besides, the MnO_2_ nanosheets coated on the surface of PFMPP NPs can catalyze the conversion of H_2_O_2_ to O_2_ (Figure 3D), alleviating hypoxia in the tumor site. Since tumor hypoxia is closely related to its invasiveness, metastasis, and recurrence, alleviating hypoxia can control tumor growth and improve the therapeutic effect to some extent. The intensity of 425 nm fluorescence intensified under higher H_2_O_2_ concentrations, which further confirmed that the PFMPP NPs have higher toxicity under the simulating tumor environment rather than in normal tissues (Supplementary Figure S5B).

To evaluate the photothermal properties of the material, we first recorded the UV-vis absorption spectrum and then tested the absorption of PFMPP NPs at the same concentration (1 mg mL^-1^). No obvious difference was found between PDA and PFMPP NPs, both showing the absorption at 808 nm. Therefore, we believed PFMPP NPs might exhibit excellent photothermal properties (Figure 3E). To verify this assumption, different concentrations of PFMPP NPs were irradiated with laser, and the respective temperature changes were temperature changes were monitored at 30-s intervals. Following 10 min of irradiation, the sample with PFMPP NPs (200 μg mL^−1^) exhibited a marked temperature rise from 23 °C to 52.8 °C, whereas DI water heated under identical conditions increased only 3 °C (Figure 3F). The heating curves of PFMPP NPs show a pronounced power-dependent increase when the laser power is raised (Supplementary Figure S6). The above results show that the photothermal conversion efficiency demonstrated linear dependence on both PFMPP NPs and laser power density. The calculated photothermal conversion efficiency of PFMPP NPs irradiated by 808 nm laser is 29.89% (Supplementary Figure S7). Thus, it can be concluded that PFMPP NPs have excellent photothermal properties under laser irradiation. Then, we tested the photothermal stability of PFMPP NPs by applying repeated rounds of laser irradiation to the solution. After four heating-cooling cycles, the maximum temperature remained stable, and the cooling time did not shorten or prolong significantly, indicating excellent photothermal stability of this material (Supplementary Figure S8). Concisely, the results showcase the great photothermal efficiency of PFMPP NPs *in vitro*, which aids the production of •OH.

### Anticancer effect of PFMPP NPs *in vitro*

**FIGURE 4:**
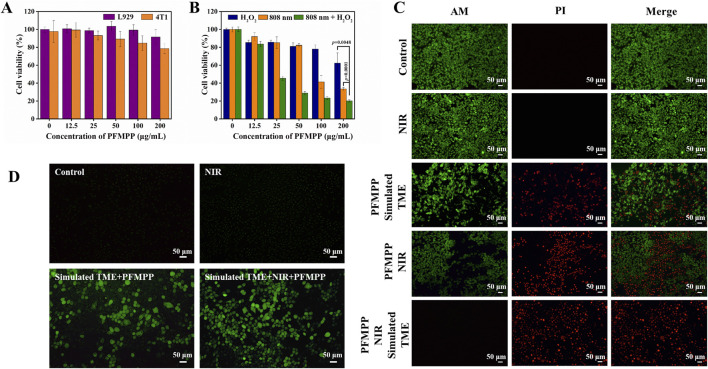
**(A)** Cytotoxicity in L929 and 4T1 cells. **(B)** CDT-only, PTT-only and CDT/PTT synergistic treatment group. **(C)** Therapeutic effect of tumor under different treatment conditions, cells viability/death imaging under different treatment conditions. **(D)** Effect of intracellular •OH production under different treatment conditions.

Inspired by the above results, we next assessed the biosafety of the PFMPP NPs *in vitro*. The cytotoxicity of PFMPP NPs was determined by CCK-8 assay. No evident influence of PFMPP NPs was found on the viability of L929 and 4T1 cells (Figure 4A). However, the cytotoxicity for 4T1 cells was slightly higher than L929, but it was still within the acceptable range, probably because 4T1 as cancer cell may have higher concentrations of H_2_O_2_ and GSH than L929. The viability of both kinds of cells was still around 80% at the highest PFMPP NPs concentration of 200 μg mL^−1^, attributed to the PAH and PAA coating. Subsequently, we used 4T1 cells to test the antitumor effect of PFMPP NPs. In CDT-only group, after co-culturing 4T1 cells with PFMPP NPs in the simulated TME, the cell survival rate decreased gradually, indicating the generation of •OH and antitumor effect by CDT. In PTT-only group, 4T1 cells were exposed to laser for 5 min, resulting in the death of tumor cells due to photothermal effect (Figure 4B). The above results demonstrate that PTT exhibited better therapeutic efficacy compared to CDT when using the same concentration of PFMPP NPs. Finally, 4T1 cells were co-cultured with PFMPP NPs in the simulated TME, and then subjected to 808 nm laser. The synergistic effect was observed for the PTT/CDT combination, which reduced cell viability to 20%.

According to the different experimental groups, 4T1 cells were divided into 5 groups and tested by living/dead cell staining. AM can bind to esterase in living cells and emit green fluorescence, while PI can pass through the cell membrane of dead cells. When PI chimerizes with DNA in the nucleus of dead cells, it emits red fluorescence. The results showed that in groups without PFMPP NPs, the cell viability after laser irradiation alone was comparable to that of the blank control, with almost no apparent cell death. Tumor cell death was observed in the individual CDT only and PTT only groups, while the combination therapy led to near-complete cell death, demonstrating a strong combined therapeutic efficacy. These results confirmed that CDT combined with PTT provided maximum tumor cell killing capability than applying the two therapies alone (Figure 4B). Furthermore, intracellular •OH generation was detected by ROS-sensitive probe DCFH-DA, which emits green fluorescence upon oxidation. In contrast to the signal of control group and NIR only group, distinct green fluorescence can be observed in PFMPP NPs only group, confirming intracellular •OH production. Notably, the green emission was substantially enhanced in cells treated with PFMPP NPs followed by 808 nm laser irradiation, supporting a photo-enhanced Fenton-like reaction that generated greater ROS levels (Figure 4D). Nevertheless, DCFH-DA probe provides indirect evidence for •OH generation, as it can be oxidized by multiple reactive species but lacks absolute specificity. After verifying that •OH can be generated intracellularly, we further examined whether PFMPP NPs could consume intracellular GSH to prevent ROS from being scavenged. As shown in Supplementary Figure S9, the intracellular GSH level in tumor cells gradually decreased with increasing concentrations of PFMPP NPs, indicating that PFMPP NPs can reduce intracellular GSH concentration and thus protect the generated ROS from excessive elimination.

### Anticancer effect of PFMPP NPs *in vivo*

**FIGURE 5:**
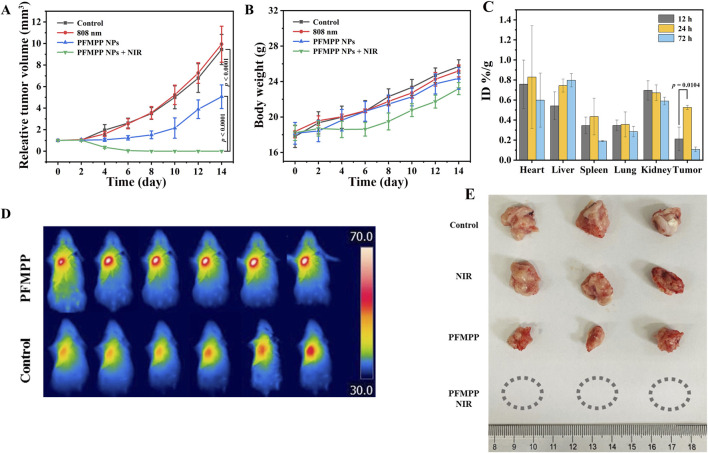
**(A)** Tumor volume changes in each treatment group over time. **(B)** Changes of body weight of mice in each group. **(C)** Distribution of PFMPP NPs (Mn) in different organs and tumor site of mice. **(D)** Temperature changes at tumor sites in mice following injection of PBS solution and PFMPP NPs. **(E)** The tumor size of each group after treatment.

Guided by the *in vitro* experimental results, we assessed the therapeutic effect of PFMPP NPs *in vivo*. From the analysis of biodistribution results, we believe that approximately 24 h after tail vein injection, the accumulation of PFMPP NPs at the tumor site reaches an appropriate level suitable for imaging and antitumor therapy (Figure 5C; Supplementary Figure S10). To prove the photothermal effect *in vivo*, the tumor-bearing mice were exposed to the 808 nm laser for 10 min after injecting with PFMPP NPs or saline via tail vein. The tumor temperature in mice treated with PFMPP NPs reached approximately 70 °C under laser irradiation. In contrast, saline-injected mice showed no notable photothermal effect under the same conditions (Figure 5D). Hence, PFMPP NPs exhibit strong photothermal activity, enabling efficient heating of the tumor site under laser exposure. Under 808 nm laser irradiation at 1.0 W cm^-2^ for 10 min, the tumor temperature reached about 70 °C, a temperature range capable of inducing local thermal ablation. It is necessary to further optimize the laser parameters to balance antitumor efficacy and tissue biosafety in follow-up study. The tumor-bearing mice were divided into four groups randomly: (I) control group, injected with saline; (II) laser-only group, irradiated with 808 nm laser; (III) CDT group, injected with PFMPP NPs; (IV) CDT/PTT group, injected with PFMPP NPs followed by NIR irradiation.

**FIGURE 6:**
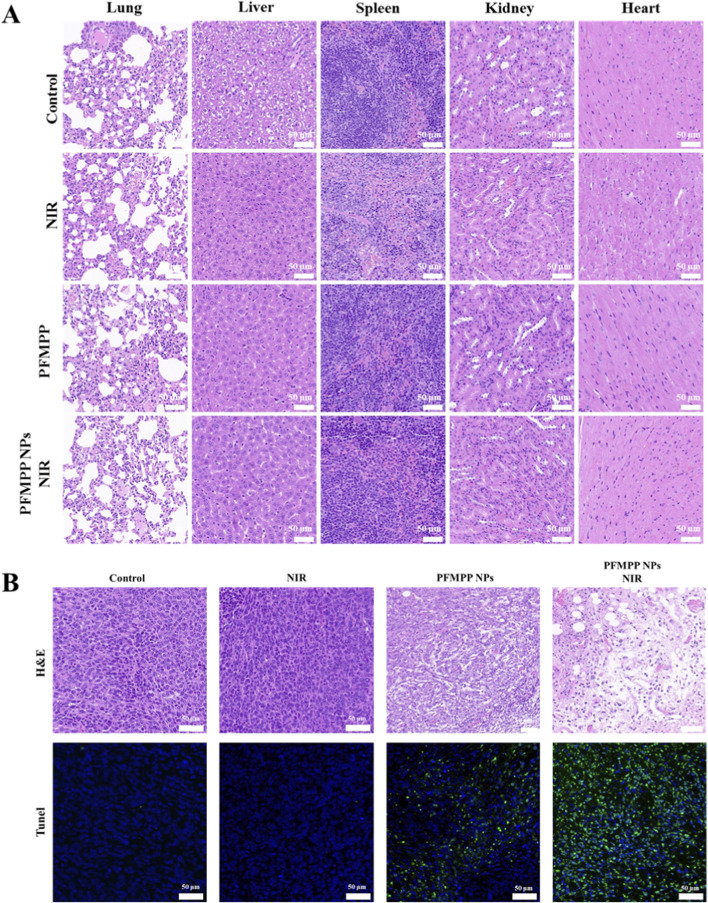
**(A)** H&E staining of important organs of mice within different treatment groups. **(B)** Apoptosis profile at the tumor site at the end of the second treatment.

After 14 days of treatment, the CDT/PTT group had an excellent antitumor effect, and lesions disappeared completely at the end of the treatments (Figure 5E). Meanwhile, the CDT group showed a significantly greater reduction in tumor volume compared to the control and laser-only groups (Figure 5A). The above results were further verified by the histological analysis of tumor cells by H&E staining and TUNEL staining. The tumor cells of (III) and (IV) experimental groups showed more severe necrosis and apoptosis (Figure 6B).

As evident from Figure 5B, throughout the 14-day treatment period, body weights remained comparable across all groups, with no significant intergroup differences. Furthermore, the results were confirmed by H&E staining (Figure 6A), which showed no significant damage of all important organs during the treatment, confirming the biocompatibility of PFMPP NPs *in vivo*. Finally, we evaluated the laboratory examination indexes of mice. The obtained results showed no considerable difference in blood biochemistry and blood routine indexes between the CDT/PTT synergistic group and the control group after 30 days, which further proved the biosafety of PFMPPNPs in tumor therapy (Supplementary Figure S11). Although our current study performed 30-day biosafety evaluation, but long-term observation is more rigorous for *in vivo* application, as emphasized in recent long-term biocompatibility studies lasting 90 days or longer (Jakic et al., 2024; Su and Kang, 2020). These studies have demonstrated that comprehensive long-term assessment of organ toxicity, degradation, and clearance is essential to confirm the biosafety of nanomaterials. Considering the experimental design, the hematology and blood biochemistry analysis in this work was performed based on single-dose intravenous injection of PFMPP NPs. Therefore, these results cannot be simply extrapolated to the long-term biosafety situation after repeated administration. Since the therapeutic regimen in this study involves multiple dosing, more systematic safety assessments matched with repeated treatment schedules will be required in subsequent research to further verify the *in vivo* biocompatibility.

### T_1_/T_2_-weighted MRI of PFMPP NPs *in vitro* and *in vivo*

As a complex core-shell structure, MnO_2_ in the outermost layer of PFMPP NPs can be used to catalyze O_2_ production and be slowly decomposed into Mn^2+^ in the special TME of acidity and high GSH. It is well known that Mn^2+^ is a paramagnetic substance with imaging ability in T_1_-weighted MRI. Besides, the Fe-BTC structure has also been proved to have T_2_-weighted MRI ability. Therefore, we tested the imaging ability of PFMPP NPs *in vitro*.

**FIGURE 7:**
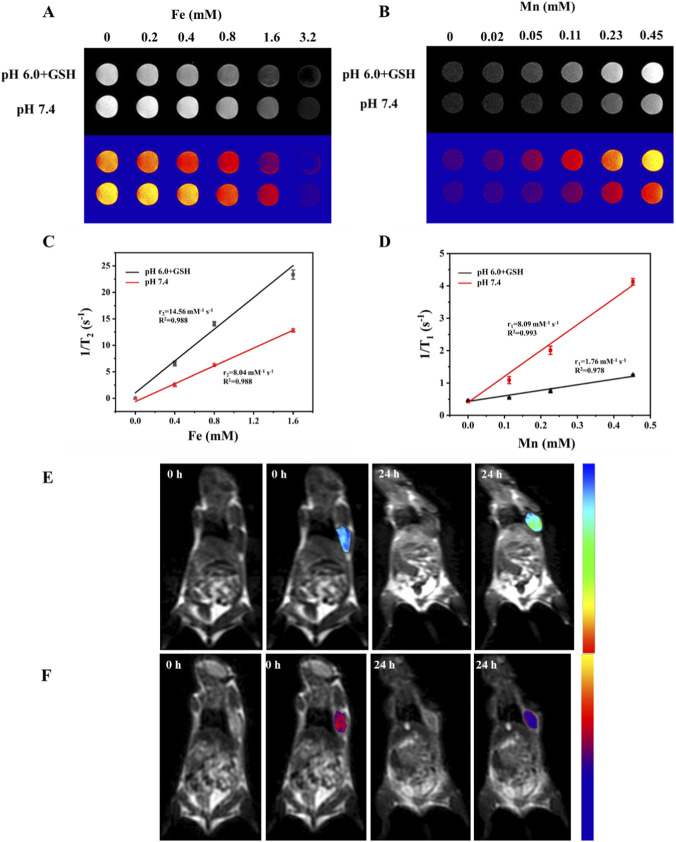
**(A)** and **(B)** MRI T_1_-and T_2_-weighted imaging and its pseudocolor map. **(C)** and **(D)** relaxivity r_1_ and r_2_ value PFMPP NPs. **(E)** and **(F)** Dual-modal imaging of PFMPP NPs in tumor site of one mouse.

*In vitro* imaging demonstrated only a minimal increase in T_1_-weighted signal intensity at pH 7.4. However, Mn^2+^ was released during the simulated tumor microenvironment condition by the decomposition of MnO_2_. Consequently, the gradual increase of Mn^2+^ concentration in the supernatant of the sample (0–0.32 mM) enhanced the T_1_ signal intensity significantly. The relaxivity r_1_ was calculated to be 8.09 mM^−1^ s^−1^ (Figures 7A,C). During T_2_-weighted imaging, the imaging ability of the material depended on the concentration of Fe^3+^, and the transverse relaxivity r_2_ was 14.56 mM^−1^ s^−1^ (Figures 7B,D). After *in vitro* experiments confirmed that PFMPP NPs have the ability of MR T_1_/T_2_ dual-modal imaging in response to TME, we further tested its imaging ability *in vivo*.

The results of tissue distribution confirmed that the concentration of nanomaterials was appropriate in the tumor site at 24 h (Figure 5D). As displayed in Figures 7E,F, the MRI signal of the tumor site on the same mouse was significantly different 24 h after injecting the PFMPP NPs via the tail vein. The T_1_-weighted imaging of the tumor became brighter, while the T_2_-weighted imaging darkened, enabling the dual-modal imaging of tumors by PFMPP NPs. Considering the limited experimental replicates in the current study, we only preliminarily verified the T_1_/T_2_ dual-modal MRI contrast performance of PFMPP NPs. Further quantitative analysis involving more animal individuals, CNR calculation and dynamic imaging comparison will be carried out in subsequent research to further confirm its dual-modal MRI advantages.

## Conclusion

In summary, we successfully synthesized a novel core-shell structure nanomaterial for TME-modulating T_1_/T_2_ dual-modal MRI guided antitumor effect via PTT and CDT combined treatment. The outermost MnO_2_ layer of this composite material can catalyze H_2_O_2_ decomposition to generate O_2_ alleviating the hypoxia of the tumor and realizing TME-modulating T_1_-weighted MRI. Moreover, the exposed Fe-BTC structure could be used as T_2_-weighted MRI contrast agent and facilitated the killings of tumor cells by generating •OH through Fenton-like reaction in the tumor site. In addition, as an excellent photothermal agent, PDA can perform PTT with the irradiation of the 808 laser. At the same time, the temperature increase further improves the treatment effect of CDT. Conclusively, PFMPP NPs integrate multiple functions such as MRI dual modal imaging, PTT and CDT combined treatment and have broad prospects in the diagnosis and treatment of solid tumors. Furthermore, pharmacokinetic analysis and long-term *in vivo* biosafety evaluation warrant our particular attention. These efforts will further enhance the clinical potential of nanomaterials in the field of tumor theranostics.

## Data Availability

The original contributions presented in the study are included in the article/Supplementary Material, further inquiries can be directed to the corresponding author.
