# Supplementary material for: Core-shell structured nanomaterials in dual-modal magnetic resonance imaging guided antitumor effect via combined treatment
Source: Front Chem. 2026 Jun 16;14:1831059. doi: 10.3389/fchem.2026.1831059 (PMC13314854; doi:10.3389/fchem.2026.1831059)
Supplement: Supplementary file 1 [file DataSheet1.pdf]

Core-shell Structured Nanomaterials in Dual-modal Magnetic Resonance Imaging guided antitumor effect via combined treatment

Wei Liu[1], Chengxin Liu[1], Longhai Jin[1], Jianqiu Wang[1], Jiale Tian[1], Tianqi Zhang[1]*, Jianhua Liu[1], Yinghui Wang[2] and Shuyan Song[2]

[1]Department of Radiology, the Second Hospital of Jilin University
[2]State Key Laboratory of Rare Earth Resource Utilization, Changchun Institute of Applied Chemistry Chinese Academy of Sciences

Characterization: Powder X-ray diffraction (XRD) was tested on a Bruker D8 Focus powder X-ray diffraction with Cu Kα radiation (λ = 1.5418 Å). Inductively coupled plasma (ICP) analyses were obtained from Varian Liberty 200 spectrophotometer. The UV−vis−NIR spectra were recorded on spectrometer (SHI-MADZU, UV-3600). Infrared thermal imaging used Fluke FLIR T420 camera. Scanning electron microscope (SEM) were performed by a FEI Qunta250 field emission scanning electron microscope. Transmission electron microscopic (TEM) images were obtained from a TECNAI G2 high resolution transmission electron microscope. X-ray photoelectron spectroscopy (XPS) spectra were performed on an ESCALAB-MKII 250 photoelectron spectrometer (VG Co.). Scanning electron microscope (SEM) were obtained from a FEI Qunta250 microscope. Fluorescence spectrum were obtained from a Hitachi F-7000 spectrometer. MRI was performed on an Ingenia 3.0T CX Magnetic Resonance. Zeta potential and dynamic light scattering (DLS) were measured on a Malvern Zetasizer Nano.

Materials: The dopamine hydrochloride, iron (III) chloride hexahydrate, Poly(acrylic acid) (PAA) (average Mv~450,000) were purchased from Aladdin Industrial Company. Trimesic acid was obtained from Macklin Company. The KMnO4, H_2O_2, ethanol, ammonia solution (30%) were purchased from Sinopharm Chemical Reagent Company. The poly(allylamine hydrochloride) (PAH) (average Mw~17,500) was obtained from Sigma Aldrich. All chemicals were analytical grade and used as received without further purification. RPMI-1640 and fetal bovine serum (FBS) were purchased from Gibco (New York, USA). The Cell Counting Kit-8 and GSH assay kits (Cellular Glutathione Peroxidase Assay Kit with DTNB) were purchased from Jiancheng Institute of Bioengineering, China. The Calcein-AM/PI (Live/Dead Cell Double Stain Kit) and DCFH-DA were purchased from Solarbio Life Sciences Company.

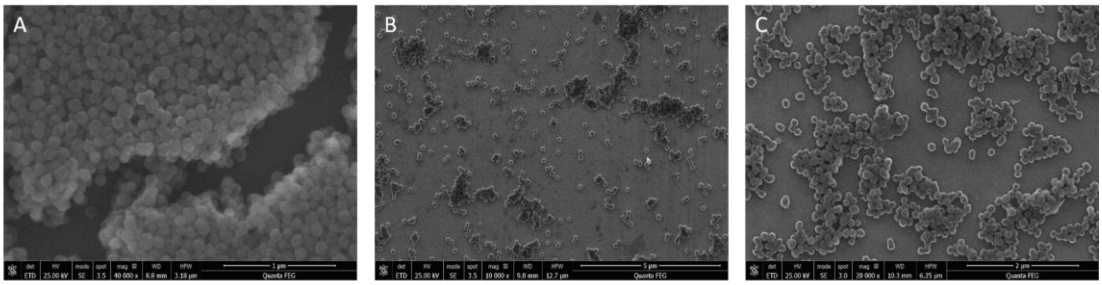

Figure S1. A) SEM image of PDA. B) SEM image of PF NPs. C) SEM image of PFM NPs.

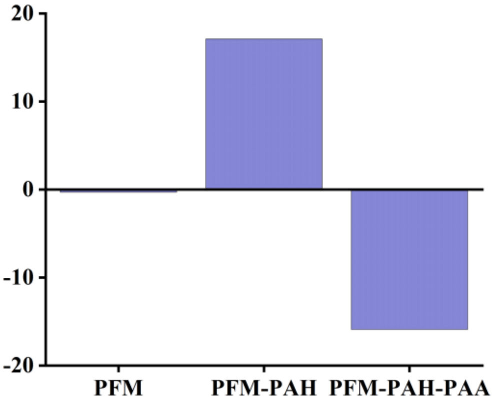

Figure S2. The surface charge changes of PFM NPs before and after modification of PAH and PAA.

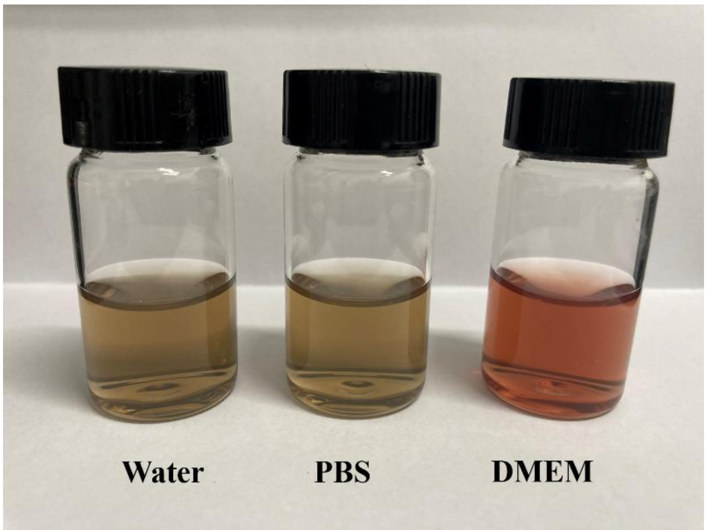

Figure S3. Dispersion of PFMPP NPs in Water, PBS solution and DMEM medium.

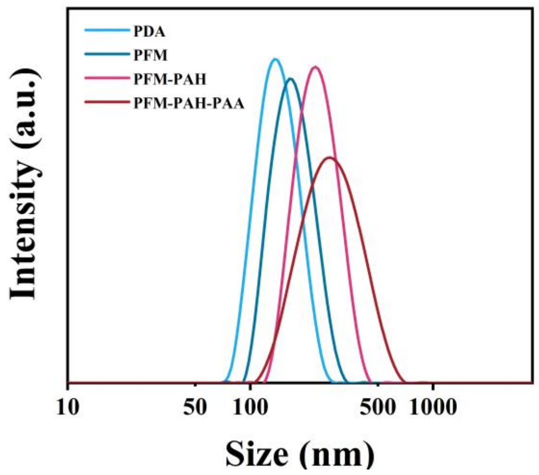

Figure S4. DLS results of nanoparticles with different steps.

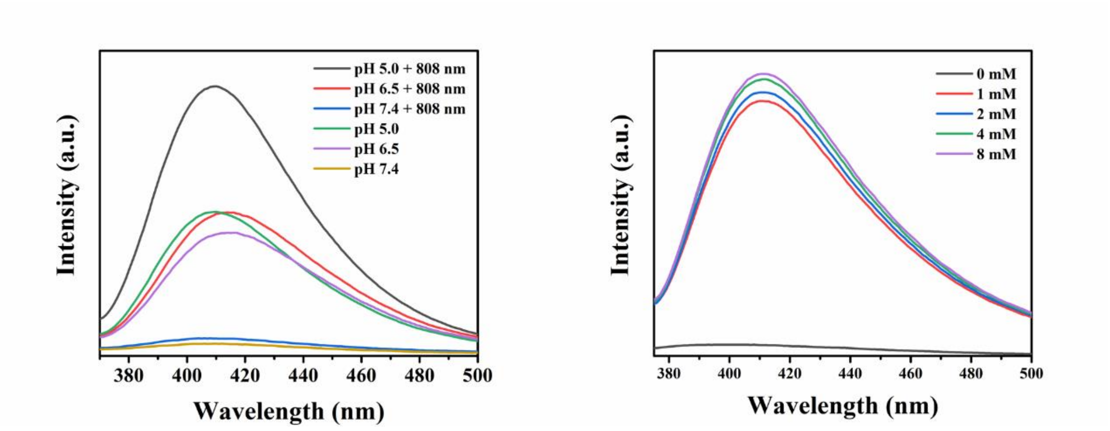

Figure S5. A) Comparison of ·OH production before and after 808 nm laser irradiation. B) The different amount of ·OH production under different concentrations of H_2O_2.

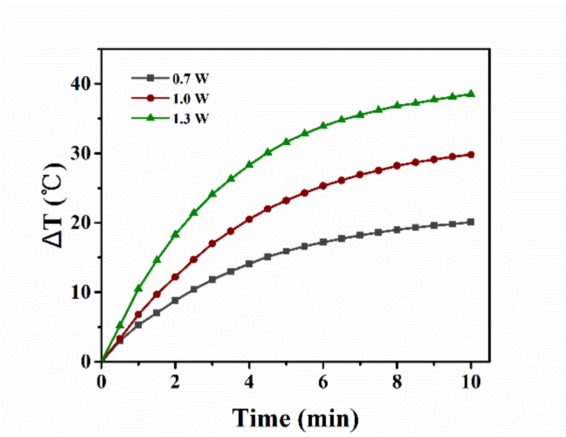

Figure S6. Temperature change of PFMPP NPs (200ug/mL) under different power of 808 nm irradiation.

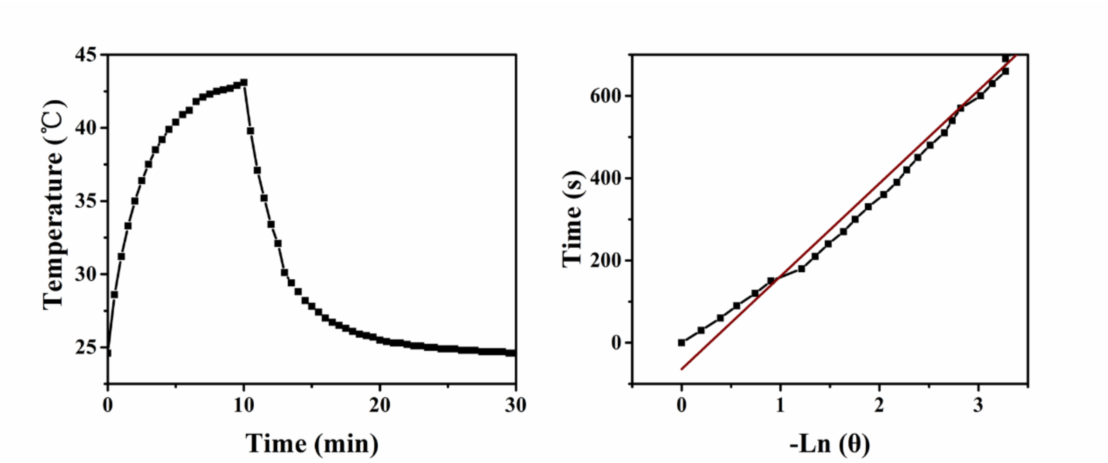

Figure S7. Plot of cooling time versus negative natural logarithm of driving force temperature.

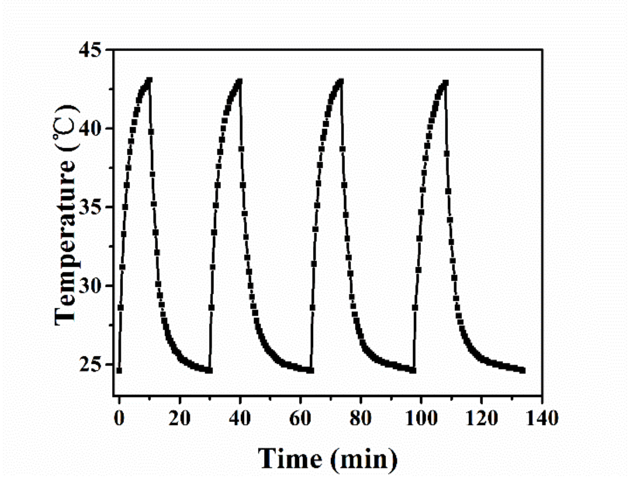

Figure S8. Photothermal stability curve of PFMPP NPs (100ug/mL) after 4 heating-cooling cycle.

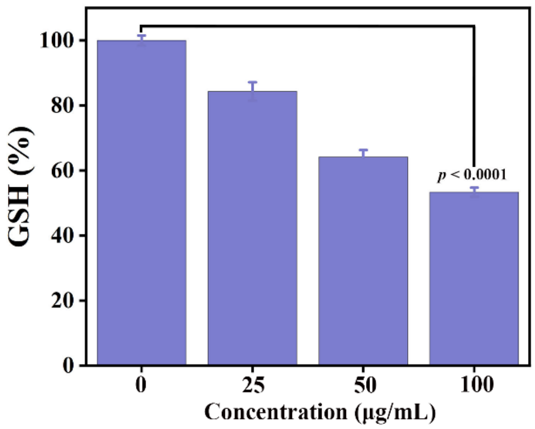

Figure S9. Percentage of intracellular GSH content under different concentration of PFMPP NPs.

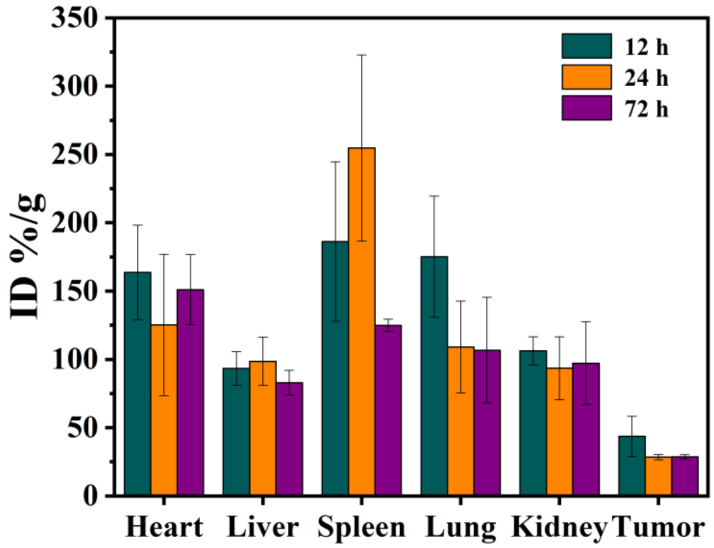

Figure S10. C) Distribution of PFMPP NPs (Fe) in different organs and tumor site of mice.

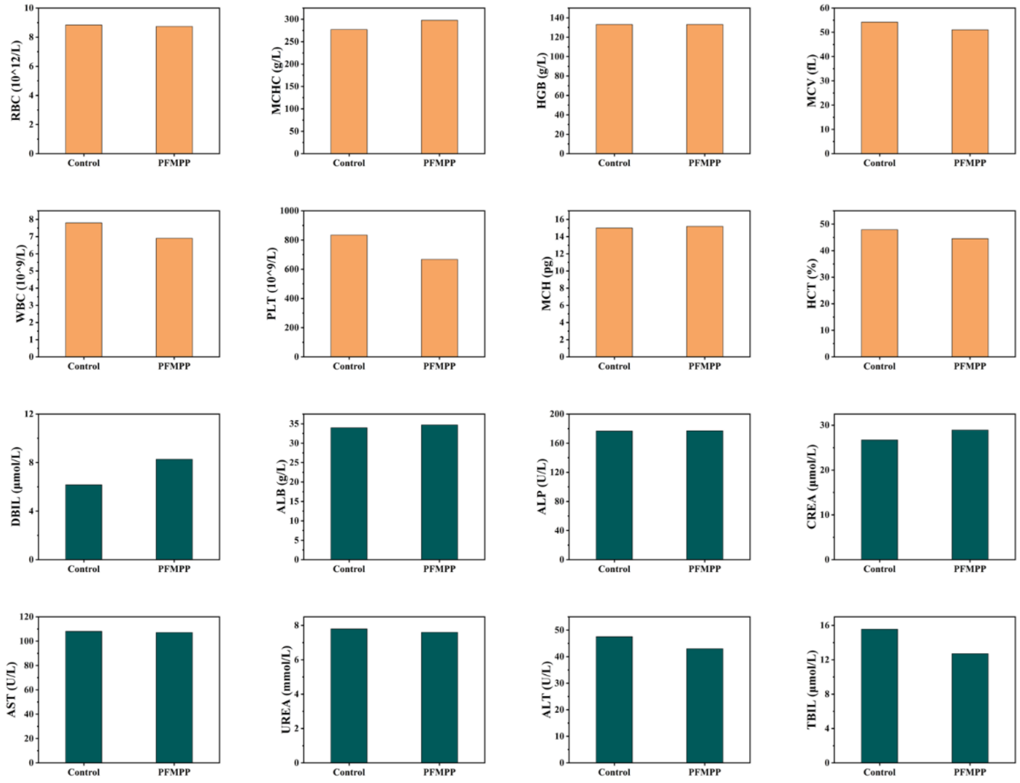

Figure S11. Hematology analysis and blood biochemistry detection results after intravenous injection of PFMPP NPs at 30 d.
